# Supplementary material for: BMP-dependent mobilization of fatty acid metabolism promotes Caenorhabditis elegans survival on a bacterial pathogen
Source: Dis Model Mech. 2025 Nov 25;18(11):dmm052357. doi: 10.1242/dmm.052357 (PMC12690539; doi:10.1242/dmm.052357)
Supplement: Supplementary information [file dmm-18-052357-s1.pdf]

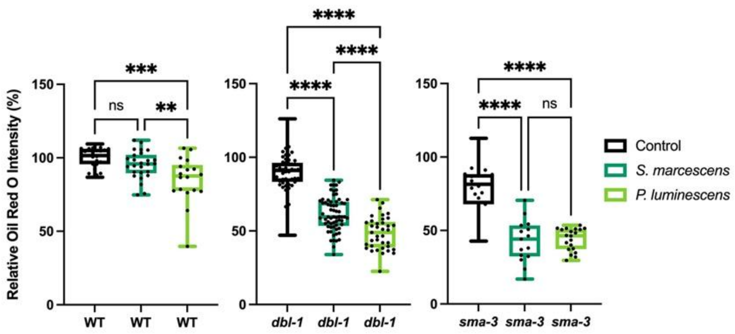

Fig. S1. Quantification of fat storage relative to wildtype control.

Lipid accumulation of wildtype, *dbl-1* and *sma-3* animals, respectively, after 24 hour pathogen exposure, stained with ORO. Quantification is presented as normalized to the wildtype control. This figure is a companion to Figure 1B,C,D, in which normalization is calculated relative to each internal genotype control. ORO experiments were repeated in triplicate on independent biological samples, with at least 15 animals per condition. Data points represent individual animals. Brown-Forsythe and Welch ANOVA multiple comparisons tests were used to determine significance.

ns = p > 0.01; * = p<=0.05; ** = p<=0.01; *** = p<=0.001; **** = p<=0.0001.

Disease Models & Mechanisms • Supplementary information

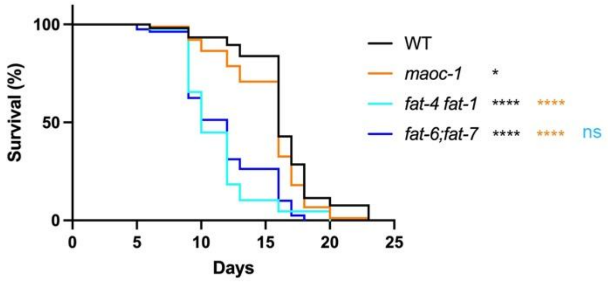

Fig. S2. Fatty acid desaturation is more involved in survival after pathogen exposure than β-oxidation.

Survival analysis of wildtype, *maoc-1*, *fat-4 fat-1* and *fat-6;fat-7* animals on *S. marcescens* bacteria. N values: WT (105), *maoc-1* (89), *fat-4 fat-1* (87), *fat-6;fat-7* (80).

ns = p > 0.01; * = p<=0.05; ** = p<=0.01; *** = p<=0.001; **** = p<=0.0001.

Black asterisks denote significance relative to wildtype control; orange is significance relative to *maoc-1*; teal is significance relative to *fat-4 fat-1*.

Table S1. Gene list of differentially expressed genes in N2 on *S. marcescens vs. E coli.*

Available for download at
https://journals.biologists.com/dmm/article-lookup/doi/10.1242/dmm.052357#supplementary-data

Table S2. Gene list of differentially expressed genes in *dbl-1* on *S. marcescens vs. E coli.*

Available for download at
https://journals.biologists.com/dmm/article-lookup/doi/10.1242/dmm.052357#supplementary-data

Table S3. Gene list of genes induced in N2 but not in *dbl-1* mutants on *S. marcescens* vs. *E coli.*

Available for download at
https://journals.biologists.com/dmm/article-lookup/doi/10.1242/dmm.052357#supplementary-data

Disease Models & Mechanisms • Supplementary information
